# Stability and Composting Behaviour of PLA–Starch Laminates Containing Active Extracts and Cellulose Fibres from Rice Straw

**DOI:** 10.3390/polym16111474

**Published:** 2024-05-23

**Authors:** Pedro A. V. Freitas, Chelo González-Martínez, Amparo Chiralt

**Affiliations:** Institute of Food Engineering FoodUPV, Universitat Politècnica de València, 46022 Valencia, Spain; cgonza@tal.upv.es (C.G.-M.); dchiralt@tal.upv.es (A.C.)

**Keywords:** biodegradable materials, phenolic compounds, cellulose fibres, valorisation of residues, bilayer films

## Abstract

The stability and composting behaviour of monolayers and laminates of poly (lactic acid) (PLA) and starch with and without active extracts and cellulose fibres from rice straw (RS) were evaluated. The retrogradation of the starch throughout storage (1, 5, and 10 weeks) gave rise to stiffer and less extensible monolayers with lower water vapour barrier capacity. In contrast, the PLA monolayers, with or without extract, did not show marked changes with storage. However, these changes were more attenuated in the bilayers that gained water vapour and oxygen barrier capacity during storage, maintaining the values of the different properties close to the initial range. The bioactivity of the active films exhibited a slight decrease during storage, so the antioxidant capacity is better preserved in the bilayers. All monolayer and bilayer films were fully composted within 90 days but with different behaviour. The bilayer assembly enhanced the biodegradation of PLA, whose monolayer exhibited a lag period of about 35 days. The active extract reduced the biodegradation rate of both mono- and bilayers but did not limit the material biodegradation within the time established in the Standard. Therefore, PLA–starch laminates, with or without the valorised fractions from RS, can be considered as biodegradable and stable materials for food packaging applications.

## 1. Introduction

Conventional petroleum-based plastics have been extensively employed in different sectors thanks to their affordability, mechanical and chemical resistance, good barrier properties, and possible reusability [1,2]. Unfortunately, the long-term stability and durability of these polymers have caused an accumulation of them in the environment, contaminating freshwater systems, cities, landfills, and seas. Likewise, the incorrect disposal of plastics has led to derived problems, such as the so-called microplastics—small particles (<5 mm) that are produced in the form of granules or originate from plastic debris [3]. Several studies have shown the harmful effects of microplastics, which can accumulate in the atmosphere, urban areas, and aquatic environments, contaminating seas, fishes, and river sediments, thus alarming the population about the potential health and social-environmental problems they can provoke [4,5]. In addition, conventional polymers are rarely used as pure material but are incorporated with additives, such as phthalates and bisphenol A, to improve their performance. These additives can be released from the polymer matrix gradually and cause health problems in animals and/or humans [5,6]. To reduce the negative effects of conventional plastics on the environment, recycling programs have been implemented, and new sustainable, biodegradable polymers have been developed to replace non-degradable plastics.

Another current challenge in terms of sustainability is waste valorisation in the context of the circular economy, which makes it possible to exploit these materials generated in different agro-industrial processes and convert them into useful resources for other purposes. Rice straw (RS) is an agro-food waste that is produced worldwide with a high potential for utilisation and valorisation since 1.5 kg of straw is generated for every kg of rice [7]. RS is usually burned after the rice harvest, which contributes to increasing air pollution due to the release of greenhouse gases, polycyclic aromatic hydrocarbons, and a variety of dioxins [8,9]. RS is a lignocellulosic residue containing about 37% cellulose, 20% hemicellulose, 20% lignin, and 17% ash. Also, RS has a high phenolic content bound to the plant matrix that can be extracted for its use in different sectors, such as food, pharmaceuticals, or cosmetics [10]. Previous studies analysed the obtention of phenolic-rich, bioactive extracts from RS using different green processes oriented to maximise the yield, using water as a solvent, such as the application of ultrasound pre-treatments or extraction with subcritical water at high temperatures and pressures [10]. The cellulose-rich extraction residue has been used to obtain cellulose fibres with many applications, including as a reinforcing agent in packaging materials and the production of cellulose aerogels [11,12,13,14].

Active extracts from agri-food waste can be used to obtain active polymeric materials for food packaging, thus extending the shelf life of food while providing a strategy for the valorisation of by-products. Active materials in food packaging interact with the food or its environment, contributing to preserving or improving the final quality of the packaged food, as well as reducing the risk of contamination [15]. Obtaining food-active packaging with biodegradable polymers is a sustainable strategy because it allows the composting of the materials since recycling them is complicated in many cases due to the organic load carried by the packaging itself. However, it is necessary to adequately match the properties of biodegradable packaging to food packaging requirements, such as high barrier capacity to water vapour and oxygen or suitable mechanical resistance, as well as to ensure their storage stability and composting capacity.

Biodegradable polymer multilayer laminates are suitable for obtaining adequate food packaging properties. The materials complement each other, giving rise to a more effective barrier against water vapour and gases that cause food spoilage [16]. Thus, laminates enhance the functional properties of the material with respect to individual layers, such as improved mechanical performance and barrier capacity [16,17]. This practice, widely used with conventional non-biodegradable plastics, presents problems for recycling due to the difficulty of separating the layers. However, this would not be a problem with biodegradable laminates, as they could be composted without layer separation. To this end, the combination of hydrophilic (high oxygen barrier) with hydrophobic (high water vapour barrier) biodegradable polymers is key to their functional performance [18].

Of the biodegradable polymers, starch is a semi-crystalline biopolymer composed of amylose and amylopectin, which are linear and branched chains of D-glucopyranose units [19]. It is a polymer with a hydrophilic nature and the ability to form thermoplastic films with a good oxygen barrier but with high water vapour permeability and water solubility [19,20]. Likewise, poly (lactic acid) (PLA) is a bio-based polymer with interesting properties for use in several applications, including food packaging. PLA is biodegradable under composting conditions and biocompatible and exhibits good water vapour barrier properties, high mechanical resistance, and photostability, although it has a limited oxygen barrier capacity [21]. Therefore, starch and PLA films are complementary materials to produce laminates with improved functional properties for food applications, where the PLA layer should be the food contact side in wet food. Muller et al. [22] prepared bilayer films based on amorphous PLA and cassava starch by incorporating cinnamaldehyde into the PLA layer, with good interlayer adhesion and a remarkable improvement of mechanical and barrier properties with respect to monolayer films. 

In a previous study, Freitas et al. [13,23] obtained PLA–starch laminates by incorporating cellulose fibres (in the starch layer) and an active extract (in the PLA layer), both obtained from RS. Laminates exhibited significantly improved barrier and tensile properties with respect to isolated films while having active properties conferred by the RS extract. These laminates were tested in their ability to preserve fresh pork meat under cold storage conditions, and a great extension of the meat shelf life was observed with respect to a control sample. Nevertheless, the stability of these materials throughout time and their composting behaviour were not analysed. It is worth mentioning that laminates must maintain the stability of their properties over time to ensure their performance throughout their commercialisation period. Likewise, it is important to validate their composting behaviour to ensure their potential for use as compostable packaging.

The aim of this study was to analyse the stability and composting behaviour of starch-PLA laminates containing or not active extracts and cellulose fibres from RS. To this end, the physical (optical, mechanical, and barrier) and active (total phenolic content and antioxidant capacity) properties of the laminates, in comparison to the corresponding monolayers, were analysed at different storage times under controlled conditions in order to evaluate potential changes in their functionality as active packaging material. Likewise, biodegradation and disintegration behaviours under composting conditions were evaluated for the different laminates and monolayers in order to learn the effect of bilayer assembly and the presence of active/reinforcing compounds on polymer degradation.

## 2. Materials and Methods

### 2.1. Chemicals

Corn starch (~27% amylose) was supplied by Roquette (Roquette Laisa, Benifaió, Spain). Amorphous PLA 4060D (average molecular weight of 106,226 D, density 1.24 g·cm^−3^) was purchased from Natureworks (Plymouth, MN, USA). Glycerol, di-phosphorus pentoxide (P_2_O_5_), acetic acid, magnesium nitrate (Mg(NO_3_)_2_), and sodium carbonate (Na_2_CO_3_) were obtained from PanReac Quimica S.L.U. (Castellar del Vallés, Spain). Poly (ethylene glycol) (PEG1000), Folin-Ciocalteau reagent (2 N), 2,2-Diphenyl-1-picrylhydrazyl (DPPH), gallic acid, methanol (>99.9% purity), and sodium chlorite (NaClO_2_) were supplied by Sigma-Aldrich (St. Louis, MO, USA).

### 2.2. Obtaining Cellulosic and Active Fractions from RS

Cellulose fibres and active extract from RS were obtained and characterised as described in previous studies [13,24]. Briefly, RS (*Oryza sativa* L.), J. *Sendra var*., from L’Albufera rice fields (“Banco de Paja”, Valencia, Spain) was dried at 50 ± 2 °C under vacuum (0.5 mmbar) for 16 h and then milled (IKA, model M20, Staufen, Germany) and sieved to obtain particles of under 0.5 mm. The extract was obtained by applying a sequential combination of ultrasound-assisted extraction and heating under reflux conditions using water as a solvent with an RS/distilled water ratio of 1:20 (*w*/*v*). A probe high-intensity ultrasonic homogeniser (Vibra CellTM VCX750, 750 W, Sonics & Material, Inc., Newton, CT, USA) was used operating at a frequency of 20 kHz, 40% sonication amplitude, at 25 °C (using an ice bath to maintain the temperature), for 30 min. After that, the aqueous dispersion was submitted to heating using a typical reflux apparatus (100 °C) for 1 h and then filtered using a qualitative filter (Filterlab, Vidra Foc, Barcelona, Spain). The active extract powder was obtained from the aqueous soluble fraction by freeze-drying at −65 °C and 0.8 mbar for 72 h. This extract had a total phenolic content (TPC) of 37.1 ± 0.4 mg gallic acid equivalent (GAE). g^−1^ of freeze-dried extract, as well as a DPPH radical scavenging capacity, expressed in terms of EC_50_, of 6.3 ± 0.3 mg freeze-dried extract·mg^−1^ DPPH [10]. The cellulose fibres were purified from the insoluble fraction of the extraction process by bleaching with sodium chlorite and characterised as to their cellulose, hemicellulose, lignin, and ashes content, which were 66, 16, 5, and 5% wt., respectively [12].

### 2.3. Film Preparation

Monolayer and bilayer films based on poly (lactic acid) (PLA), with and without active extract, and thermoplastic starch, with and without cellulose fibres, were prepared according to Freitas et al. [13].

#### 2.3.1. TPS Monolayers

Thermoplastic starch films, with (TPScf) or without (TPS) cellulose fibres at 3% wt. with respect to the total polymer mass, were obtained by melting blending and compression moulding process using glycerol as a plasticiser at 30% wt with respect to the polymer. Pre-conditioned corn starch (P_2_O_5_ at 25 °C for 3 days) was firstly mixed with the other film components and then melt-blended in an internal mixer (HAAKETM PolyLabTM QC, Thermo Fisher Scientific, Karlsruhe, Germany) at 130 °C and 50 rpm for 10 min. For each treatment, the obtained solid mixture was cold-milled (IKA, model M20, Germany) and conditioned in a desiccator with a Mg(NO_3_)_2_ over-saturated solution (53% RH) at 25 °C for one week. Thereafter, the films were obtained through the thermocompression of 4 g of conditioned pellets onto Teflon sheets with a hydraulic press (Model LP20, Labtech Engineering, Samut Prakan, Thailand) by applying preheating at 160 °C for 3 min, compression at 30 bar and 160 °C for 2 min, followed by 130 bar for 6 min (160 °C), and finally cooling to 70 °C. All obtained monolayer films were conditioned at 53% RH and 25 °C before their characterisation or use to obtain the bilayers.

#### 2.3.2. PLA Monolayers

Prior to preparing the films, amorphous PLA pellets were vacuum-dried at 0.5 mmbar and 60 °C for 4 h in order to eliminate residual water. To prepare the active films with RS extract (PLAes), the dried PLA pellets were hand-mixed with 6% active extract and 8% PEG1000 as a plasticiser, with respect to the polymer mass, and then melt-blended in the internal mixer at 160 °C and 50 rpm for 6 min. Control PLA films without extract were also prepared under the same conditions. The PLA and PLAes films were obtained through the thermocompression of 3 g of milled solid blend by applying preheating at 160 °C for 3 min, compression at 100 bars at 160 °C for 3 min, and finally cooling to 70 °C.

#### 2.3.3. Bilayer Preparation

The bilayer films were obtained in accordance with Freitas et al. [13] by thermocompressing the PLA or PLAes monolayers and the TPS or TPScf monolayers with all possible combinations. The films were laminated by using a hydraulic press operating at 120 °C for 2 min preheating, compression at 10 bars and 120 °C for 3 min, and finally cooling to 70 °C. The obtained bilayer films were conditioned at 25 °C and 53% RH before characterisation.

### 2.4. Characterisation of the Films

To determine the stability of the monolayer and bilayer films, all formulations were characterised according to their functional properties described below at 1, 5, and 10 weeks of storage at 25 °C and 53% RH.

#### 2.4.1. Optical Properties

A spectrocolorimetre (CM-3600d, Minolta Co., Tokyo, Japan) was used to obtain the optical properties of the films according to the Kubelka–Munk theory of multiple scattering. The film reflectance (*R*), obtained on black (*R*_0_) and white (*R_g_*) backgrounds in the range of 400 to 700 nm, was determined and then used to obtain the internal transmittance (*T_i_*) and infinite reflectance spectra (*R_∞_*), as described by Freitas et al. [12]. The CIEL*a*b* colour coordinates of the films were determined by considering the D65 illuminant and 10° observer from the *R_∞_*. Then, values of Chroma (C_ab_*) (Equation (1)) and hue angle (h_ab_*) (Equation (2)) were obtained.

(1) $$C_{ab}^{*}=\sqrt{a^{*2}+b^{*2}}$$

(2) $$h_{ab}^{*}=arctg\left(\frac{b^{*}}{a^{*}}\right)$$

#### 2.4.2. Tensile Properties

A universal test machine (TA.XTplus model, Stable Micro Systems, Haslemere, UK) was used to analyse the tensile strength vs. Henky deformation curves, and the tensile strength at break (TS), elastic modulus (EM), and elongation at break (E) were obtained, in accordance with the ASTM D882 method [25]. A digital micrometre (Palmer, model COMECTAT, Barcelona, accuracy of 0.001 mm) was used to obtain the thicknesses of the films, which were measured at ten random film positions. The tensile properties were evaluated in eight replicates for each formulation.

#### 2.4.3. Barrier Properties

The gravimetric method ASTM E96/E96M [26], modified by McHugh et al. (1993), was used to determine the water vapour permeability (WVP) of the films. Conditioned film samples (25 °C and 53% RH) of 3.5 cm diameter were placed on Payne permeability cups (Elcometer SPRL, Hermelle/s Argenteau, Belgium) filled with 5 mL of distilled water. The system was placed into desiccators containing Mg(NO_3_)_2_ (53% RH) at 25 °C and weighed periodically for 48 h. The WVP of the films was determined from the slope of the weight loss–time curves. For each formulation, the analysis was performed in triplicate.

An oxygen permeation analyser (Model 8101e, Systech Illinois, IL, USA) was used to determine the oxygen permeability (OP) of the films according to the ASTM D3985-05 method [27]. The analysis was performed at 25 °C and 53% RH, and the oxygen transmission rate (OTR) was measured until the equilibrium was reached. Each formulation was analysed in duplicate.

#### 2.4.4. Bioactive Properties

The stability of the active compounds incorporated into the films was characterised in terms of total phenolic content (TPC) and antioxidant capacity of released compounds from the films in methanol. To this end, samples of each active film (PLAes, PLAes-TPS, and PLAes-TPScf) were cut (1 mm × 1 mm) and immersed in methanol at a film/solvent ratio of 1:10 (*w*/*v*) and kept under stirring at 20 °C for 24 h to release the phenolic compounds from the polymer matrix. Afterwards, the dispersion was filtered (Filterlab, 0.45 µm, Vidra Foc, Barcelona, Spain), and the liquid fraction was characterised according to their TPC and DPPH radical scavenging capacity. The TPC was obtained using the Folin–Ciocalteu method in accordance with Freitas et al. [10] in triplicate, and the results were expressed as a percentage of phenolics released with respect to the initially incorporated in the thermoprocessed film with the active extract. The antioxidant capacity of the active compounds released was determined according to the DPPH (2,2-Diphenyl-1-pikryl-hydrazyl) radical scavenging assay [28] and expressed as mg of extract·mg^−1^ DPPH [10]. These analyses were carried out in duplicate for each formulation.

### 2.5. Composting Properties of the Films

#### 2.5.1. Compost Conditioning and Preparation of Synthetic Solid Residue

The disintegration and biodegradation tests were carried out using a ripe compost, which was prepared by removing any inert material and then sieving it (particles of under 1 cm). The pH of the ripe compost was determined in triplicate by mixing and stirring one part of the compost with five parts of distilled water.

For the disintegration test, the synthetic solid residue (SSR) was prepared following the ISO 20200 International Standard method [29], which consisted of a manual mixture of the ripe compost with different components, typically corn starch, rabbit food, corn germ oil, sucrose, urea, and sawdust. The water content of the ripe compost and the SSR was adjusted to 55% wt. by incorporating distilled water and then manually mixing the resulting material.

The ripe compost and *SSR* were also characterised for dry (*DS*) and volatile (*VS*) solids at the beginning and end of the composting process, and both were expressed in percentage. *DS* was determined by drying the samples in an oven at 105 °C until constant weight was achieved (Equation (3)), while *VS* was obtained by calcining the dry samples at 550 °C until constant weight was achieved (Equation (4)). Both measurements were carried out in triplicate.

(3) $$DS\;\left(\%\right)=\frac{W_{d}^{105}}{W_{i}}\times 100,$$

(4) $$VS\;\left(\%\right)=\frac{W_{d}^{105}-W_{d}^{550}}{W_{i}^{150}}\times 100,$$

where *W_i_* is the initial weight of the samples (ripe compost or SSR), *W_d_*^105^ is the weight of the samples after drying at 105 °C, and *W_d_*^550^ is the weight of the samples after calcining at 550 °C.

#### 2.5.2. Disintegration Test

The degree of disintegration of the films was evaluated on a laboratory scale using an adapted method from ISO 20200 (2023) [29]. Film samples were cut in 25 × 25 mm^2^, put in mesh bags (1 mm mesh size), and placed into reactors with 1 kg of SSR in each. The reactors had a hole (5 mm diameter) on the wide sides to favour gas exchange and avoid anaerobiosis. Four reactors per formulation were stored in a chamber at 58 °C to ensure thermophilic composting conditions for 73 days. The reactors were periodically weighed throughout the test, and distilled water was added to maintain the required moisture conditions if necessary. Periodic sampling for weighing and morphological analysis was carried out only in one of the reactors, where the film samples (20 per reactor) were introduced individually in the mesh bags for easy sampling at each time. On each sampling day, three individual film bags per each formulation were taken out of the reactors, cleaned with a soft brush, and then characterised according to their weight loss and appearance. After 73 days of incubation, the mesh bags containing the films were cleaned to remove adhered compost residues, vacuum-dried at 40 °C, and then weighed to determine weight loss. The percentage of disintegration of the films (*D*_73_) was calculated according to Equation (5).

(5) $$D_{73}\;\left(\%\right)=\frac{m_{i}-m_{r}}{m_{i}}\times 100,$$

where *m_i_* is the initial dried mass of the film samples, and *m_r_* is the dried mass of the residual sample at each time.

#### 2.5.3. Biodegradation Test

The aerobic biodegradation of the films under controlled composting conditions was evaluated according to the adapted method from EN ISO 14855-1:2012 European Standard [30]. This is a respirometric test based on measuring the CO_2_ generated in the biodegradation process. The CO_2_ generated is proportional to the percentage of biodegradation of the material analysed. Thus, ripe compost with 55% wt. of water was mixed with powdered film samples (2 × 2 mm^2^) at a compost/film ratio of 6:1 (*w*/*w*). The mixture was put into a polypropylene flask (60 mL) and then placed in a glass reactor (2 L capacity) with a septum in the lid. A second flask containing distilled water was also placed inside the reactor to ensure 100% RH. Reactors containing microcrystalline cellulose (CMC) as reference material mixed with ripe compost and reactors with just ripe compost as a control were also prepared. The reactors were closed and incubated in ovens at 58 ± 2 °C for 90 days. The CO_2_ produced in each reactor throughout the biodegradation process was determined using an analyser Dansensor (PBI Dansensor, CheckMate 9900 O2/CO2, Ringsted, Denmark). Once the measurement was taken, the reactors were left open for 15 min to avoid anaerobiosis conditions. The measurements were taken at different composting times: every 12 h at the beginning of the test and every 24 h at longer times. The oxygen concentration was monitored at each time to ensure that it was above 6%, which corresponded to aerobic conditions. Once a week, water was added if necessary, and the compost was gently stirred.

In order to obtain the amount of carbon equivalent of each sample, the elemental composition of the films was determined using Elemental Microanalyser (Eager 200, Micro TruSpec from LECO Instrumentos S.L., Madrid, Spain). This analysis was performed in duplicate. Considering that all the carbon present in the samples is transformed into CO_2_, the maximum theoretical amount of CO_2_ (*CO*_2_*^Th^*) that could be generated was calculated using Equation (6):

(6) $$CO_{2}^{Th}=DW_{s}\times C_{s}\times \frac{Mw_{CO_{2}}}{Mw_{C}},$$

where *DW_s_* is the dry weight of the sample (g), *C_s_* is the percentage of carbon in the sample (%), $Mw_{CO_{2}}$ is the molecular weight of CO_2_ (g·mol^−1^), and *Mw_C_* is the atomic weight of carbon (g·mol^−1^).

For each formulation, the percentage of biodegradation of the films (*B*) was determined using Equation (7) in terms of the accumulative amounts of CO_2_ generated by the sample (Ʃ*CO*_2*s*_) and corrected using the blank values (Ʃ*CO_2B_*) and the maximum theoretical amount of CO_2_ generated by the sample (*CO*_2_*^Th^*). To calculate the total mass of CO_2_ generated from the volume percentage determined in the flask headspace, this was multiplied by a factor of 3.12, corresponding to the product of the headspace volume (1965 mL) and the density of CO_2_ at 58 °C (0.00159 g·mL^−1^).

(7) $$B\left(\%\right)=\frac{\sum CO_{2_{S}}-\sum CO_{2_{B}}}{CO_{2}^{Th}}$$

In order to describe the biodegradation kinetics of the film, Hill’s model was fitted (Equation (8)) to the experimental data using Origin software (version 2020, OriginLab Corporation, Northampton, MA, USA).

(8) $$B\;\left(\%\right)=B{\%}_{max}\times \frac{t^{n}}{k^{n}+t^{n}}$$

where *B%_max_* is the percentage of biodegradation at infinite time, *t* is the time (days), *k* is the time at which 0.5 B%_max_ has occurred, and *n* is the curve radius of the sigmoid function.

### 2.6. Statistical Analysis

Multifactorial analysis of variance (MANOVA) was carried out to analyse the effect of storage time and formulation on the functional properties of the films using the least significant difference (α) of 5%. The data were analysed by using the Minitab statistical software (version 17).

## 3. Results and Discussion

### 3.1. Changes in Physical Properties of the Films during Storage

#### 3.1.1. Barrier Properties

**Figure 1:**
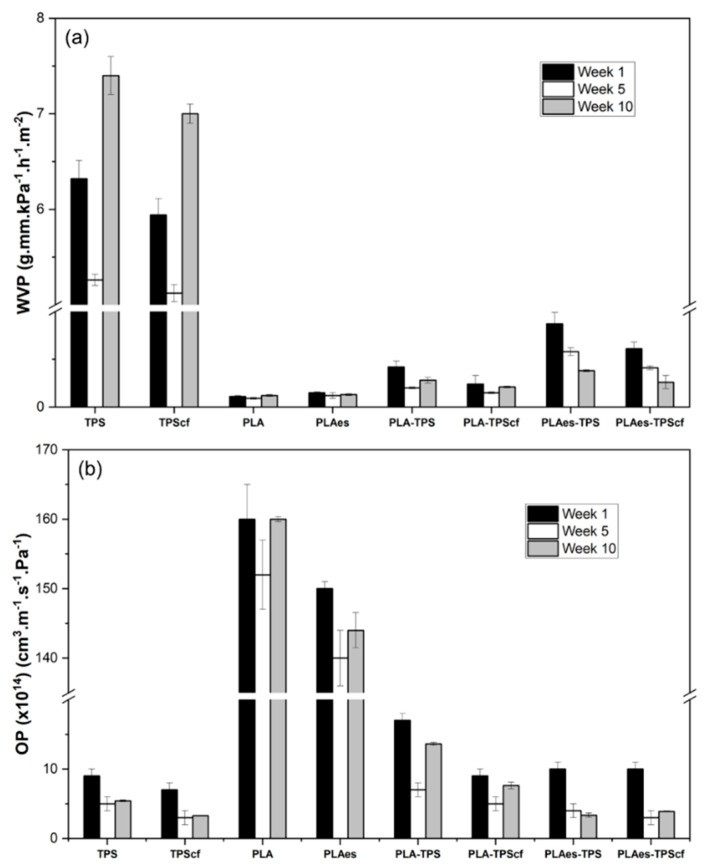
Water vapour permeability (WVP) (**a**) and oxygen permeability (OP) (**b**) of monolayer and bilayer films stored at 25 °C and 53% relative humidity for 1, 5, and 10 weeks.

The oxygen (OP) and water vapour (WVP) permeabilities of monolayers and bilayers after 1, 5, and 10 weeks of storage at 25 °C and 53% RH are shown in Figure 1. PLA monolayers exhibit high oxygen permeability but high water vapour barrier capacity, while the opposite behaviour was observed for starch films, according to their respective hydrophobic and hydrophilic nature. The obtained values of WVP and OP of the PLA and starch films were in the range reported by other authors [14,22,31]. The barrier capacity of starch monolayers to both water vapour and oxygen was enhanced by the incorporation of fibres (*p* < 0.05), whereas the incorporation of RS extract in the PLA monolayer (PLAes) slightly increased the WVP values but reduced the OP values, as observed in a previous study [21]. These effects can be attributed to different factors, such as the changes in the chemical affinity of the polymer (PLA) when polar extracts were incorporated and the potential oxygen scavenging effect of the extract antioxidants, as well as the increase in the tortuosity factor for mass transfer when cellulose fibres are present in the starch films [14].

In the bilayer assemblies, a remarkable improvement in the overall barrier capacity was observed in comparison to monolayer films due to the complementary role of each layer in limiting the transfer of water and oxygen molecules. This reflects the advantage of laminates based on materials with complementary barrier properties. The bilayers exhibited WVP values in the range of those of PLA films and OP values closer to those of starch films, which is in agreement with the lamination theory [32]. The incorporation of the extract into PLA films provoked a slight increase in the WVP values of PLAes-TPS and PLAes-TPScf bilayers with respect to the expected values. This effect was higher for WVP than for OP, suggesting greater changes in the PLA limiting layer for water transfer during the thermoadhesion step. The phenolic acids present in the RS extract could promote partial hydrolysis of PLA chains during thermoprocessing and thermocompression with starch films, which contain bound water. This would alter the interchain forces of the polymeric network, favouring the diffusion of water vapour through the films. In terms of oxygen barrier, the presence of extract (in PLAes-TPS) or cellulose fibres (in PLA-TPScf) in the bilayers decreased OP values (about 41% and 46%, respectively), similarly to that occurred in monolayers, but no additive effect was observed for the PLAes-TPScf bilayer.

The study of potential changes in the film properties throughout the storage, associated with structural polymer rearrangements, is relevant in the development of food packaging materials since their stability is necessary to ensure the preservation of packaged food. In this sense, the functional properties of the mono- and bilayers were also evaluated after 5 and 10 weeks of storage. As shown in Figure 1, the storage time significantly affected the permeability of the films, mainly in starch monolayers and bilayers, whereas PLA monolayers exhibited more stable values. These changes can be attributed to structural modifications in the polymer matrices during storage that can be related to the reorganisation of the polymer chains in the amorphous phase and recrystallisation in starch films, as well as to the interlayer compound diffusion in the laminates. The recrystallisation or retrogradation of starch occurs when the amylose chains, at a higher rate than those of amylopectin, aggregate through the double helices, forming crystalline zones stabilised by hydrogen bonds [33]. As a consequence of these chain rearrangements, the structure of the starch films becomes more compact, reducing the molecular diffusion of both water and oxygen molecules through the films. The crystallisation process releases water molecules into the amorphous phase, thus contributing to its plasticisation, which can promote water diffusion through this phase for the longest time, as observed in Figure 1. The incorporation of cellulose fibres could affect the starch retrogradation rate, promoting crystallisation since the reinforced monolayer (TPScf) presented a greater reduction in OP value than the control TPS film (57% vs. 44%).

PLA chain reorganisation also occurred over time (physical ageing) but at a lower extent than in starch. This process is a natural phenomenon that affects the physical properties of the amorphous phase of semi-crystalline polymers. The physical ageing of polymers, such as PLA, occurs at temperatures close to the glass transition temperature (T_g_) and is characterised by a decrease in specific volume, specific enthalpy and entropy, and molecular mobility. This process can affect the functional characteristics of the polymers, especially the mechanical properties of the films [34]. The laminates reflected the changes that occurred in both starch and PLA layers, as well as the potential interlayer diffusion of low molecular weight compounds, such as plasticisers (water, glycerol, and PEG) or extract compounds in the active formulations. Thus, it is remarkable that extract-free laminates exhibited a reduction in both WVP and OP after 5 weeks of storage and a subsequent increase after 10 weeks of storage, which could be mainly due to the retrogradation effect in the starch layer. However, in the extract containing laminates, a progressive reduction in both WVP and OP could be observed, which could also be associated with the progressive migration of active compounds and plasticisers between layers that can modify the initial properties of the films and their ageing behaviour. Specifically, water or glycerol migration from the starch layer to the PLA sheet may greatly affect its ageing behaviour, as described by Acioli-Moura and Sun [35], promoting the molecular mobility of PLA chains and facilitating chain aggregation.

Therefore, the lamination of starch and PLA highly improved the overall barrier properties of the material, while the incorporation of cellulose fibres and active extract promoted the oxygen barrier capacity. The storage time modifies the barrier capacity of the laminates. Nevertheless, in every case, the time positively affected the barrier properties of the films, reducing the values of OP and WVP in line with the structural modifications in the polymer layers. Thus, from the point of view of barrier capacity, both reinforced and active bilayers maintained suitable properties to be applied for food packaging in order to maintain food quality and safety and extend shelf life.

#### 3.1.2. Tensile Properties

**Figure 2:**
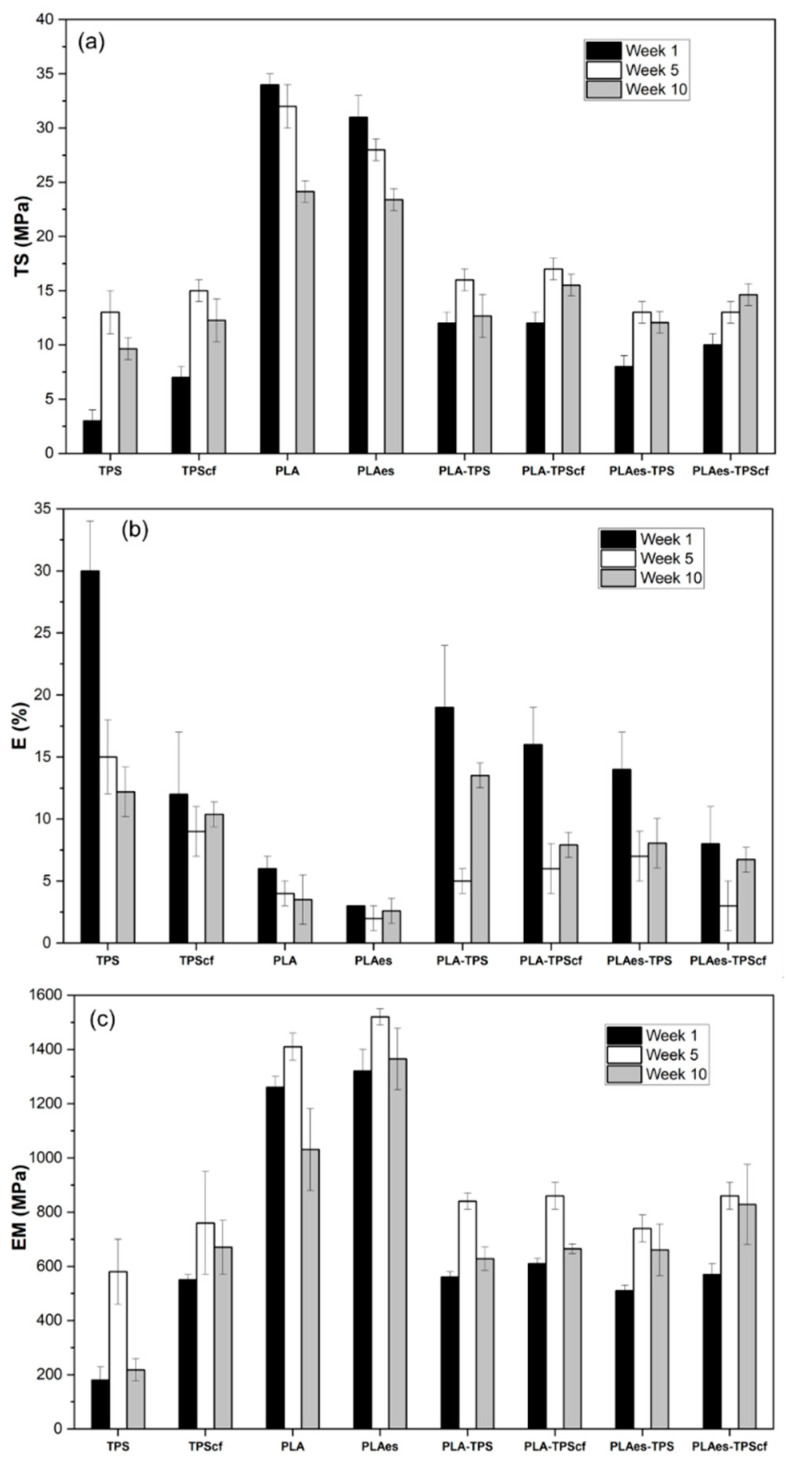
Tensile properties, namely tensile strength at break (TS) (**a**), elongation at break (E) (**b**), and elastic modulus (EM) (**c**) of monolayer and bilayer films stored at 25 °C and 53% relative humidity for 1, 5, and 10 weeks.

Figure 2 shows the mechanical properties of monolayer and bilayer films after 1, 5, and 10 weeks of storage. At one week of storage, the TS, E, and MS values of starch and PLA monolayers were in the range reported in previous studies [14,36,37]. The presence of active extract negatively affected the mechanical properties of PLA monolayers (*p* < 0.05). The PLAes film was 10% and 50% less resistant to break and stretchable, respectively, than the control PLA monolayer. This is due to molecular interactions between the extract components (mainly phenolic compounds and carbohydrates) and the PLA matrix, leading to a reduction of PLA interchain forces. The phenolic acids present in the extract can hydrolyse the PLA chains, which also contributes to weakening the polymer matrix. In contrast, the cellulose fibres enhanced the stiffness of the starch films and their resistance to break, as previously reported by other authors [14,38].

In general, all the bilayers were noticeably less resistant and stiffer than the PLA monolayers, which is the most resistant layer but gained extensibility with respect to PLA, which is the least extensible layer. As commented on above for barrier properties, these changes can be attributed to the interlayer migration of compounds, mainly during the thermoadhesion step when polymers were at a high temperature, which promotes molecular diffusion. Partial hydrolysis of PLA chains promoted by water diffusion from the TPS layer during the thermoadhesion step could affect their mechanical strength, thus worsening the mechanical behaviour of the resultant bilayer. Lim et al. [38] reported that moisture content in PLA resin above 250 ppm can lead to severe hydrolytic degradation during heat treatment. The active bilayers (PLAes-TPS and PLAes-TPScf) were the least resistant to break and extensible (*p* < 0.05), suggesting that phenolic acids in the extract could also contribute to partial hydrolysis in the PLA matrix. Muller et al. [22] also reported a decrease in the TS and EM values in the PLA–starch bilayers with respect to the expected values from the PLA layer.

After 5 weeks, the starch monolayers (TPS and TPScf) and bilayers showed a significant increase in EM and TS values, while E values decreased, coherently with the above-described process of starch retrogradation, which implies an increase in the matrix toughness in line with the amylose crystallisation. TPS films, compared with TPScf, showed the greatest changes in EM (220% vs. 38%), TS (330% vs. 114%), and E (50% vs. 25%), which suggest the greatest progress of retrogradation in fibre-free starch films. However, a significant decrease in EM occurred after 10 weeks of storage, which could be associated with the progressive plasticisation of the amorphous phase due to the water released from crystalline domains, as deduced from the development of barrier properties. This behaviour was also reflected in the laminates, according to the similar phenomenon that occurred in the starch sheet.

The PLA monolayers also exhibited a moderate increase in stiffness after 5 storage weeks, this being higher when it contains extract (15% vs. 12%) due to the physical ageing of the polymer, with a decreasing tendency of the film extensibility and resistance to break. The presence of the extract in the PLA matrix may increase the chain molecular mobility, enhancing their reorganisation to reach a higher degree of matrix compaction. However, a remarkable decrease in stiffness and resistance to breaking occurred after 10 storage weeks, which could be associated with the progressive hydrolyses of the polymer exposed to ambient relative humidity (53%). These changes were also reflected on the bilayer films and overlapped with the main changes that occurred in the starch sheet. Every laminate exhibited similar tensile properties after 10 storage weeks, but the extract and fibre-free laminates were the most extensible. All laminates had acceptable mechanical properties after 10 storage weeks for their use as flexible packaging for food applications.

#### 3.1.3. Optical Properties

**Figure 3:**
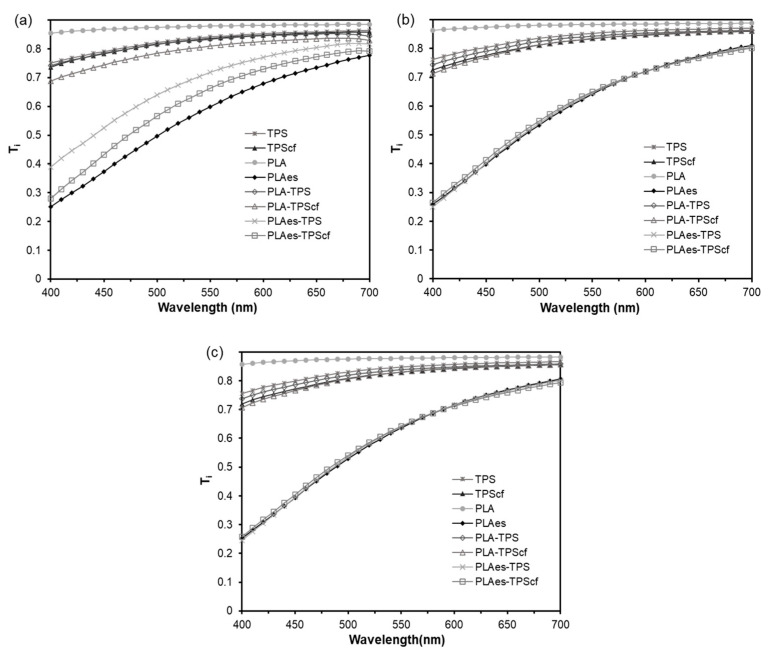
Internal transmittance spectra of monolayer and bilayer films stored at 25 °C and 53% relative humidity for 1 (**a**), 5 (**b**), and 10 (**c**) weeks. The visual appearance of the bilayers is also shown.

Figure 3 shows the visual appearance of the four bilayers and the internal transmittance (*T_i_*) spectra of all mono- and bilayer formulations (400–700 nm) after 1, 5, and 10 storage weeks. Likewise, the values of colour coordinates (L*, C_ab_*, and h_ab_*) of the films at different storage times are given in Table 1.

Concerning the appearance of the films, the main change was produced when the RS extract was incorporated into the PLA layer. The RS extract provides the PLA films with a dark brown shade, as well as the light scattering effects produced by the small dispersed particles in the matrix [21]. In fact, PLAes films exhibited the lowest L* and h_ab_* values and the highest colour saturation (C_ab_*). The PLA monolayer was the most transparent film, exhibiting the highest *T_i_* and L* values, according to the high degree of homogeneity and transparency of the polymer matrix. No significant changes in the *T_i_* or colour coordinates of the reinforced starch monolayer (TPScf) in comparison to the control film (TPS) were observed. Similar results were found by Fourati et al. [37] and Benito-Gonzalez et al. [39] for starch films reinforced with different cellulosic fractions. The bilayers exhibited the coupling of optical properties of the constituent monolayers. The bilayers with extract (PLAes-TPS and PLAes-TPScf) had optical properties closer to the PLAes film since the extract had a great influence on the *T_i_* and colour parameters. Likewise, the presence of fibres in the starch layer (PLAes-TPScf) reduced the sample internal transmittance (*T_i_*) and lightness to a greater extent than when these were not present (PLAes-TPS), leading to a redder and more saturated colour. Nevertheless, the starch layer attenuated the colour effects of the RS extract in the laminates mainly when it did not contain fibres, which promoted a light reflection of the material with the consequent selective absorption of the extract compounds. Indeed, the PLA-TPScf bilayers were less transparent than PLA-TPS, exhibiting a darker and more saturated colour.

By analysing the effect of storage time, it was observed that the colour coordinates of all films did not significantly change after 5 and 10 weeks. This indicates a certain stability of the compounds responsible for the colour in the films. Likewise, both PLA and starch monolayers and bilayers without active extract showed no change in their *T_i_* spectra after 5 and 10 weeks of storage. However, the storage time produced a decrease in the light transmittance of the active bilayers (PLAes-TPS and PLAes-TPScf), reaching a similar spectrum to the PLAes monolayer, which suggests a reduction in the attenuating effect of the starch layer on the absorbance reduction caused by the extract. This could be attributed to the migration of coloured compounds to the starch layer, thus reaching a more homogenous distribution.

#### 3.1.4. Bioactive Properties

**Figure 4:**
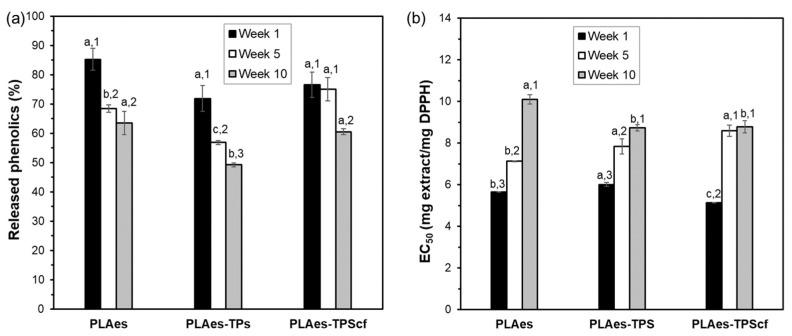
Percentage of phenolic content released from the films with respect to those initially incorporated with the extract (**a**) and the antioxidant capacity of the extract present in the films (PLAes monolayer and PLAes-TPS and PLAes-TPScf bilayers) (**b**) stored at 25 °C and 53% relative humidity for 1, 5, and 10 weeks. Different letters indicate significant differences between the formulations at constant time. Different numbers indicate significant differences between the storage times for each formulation.

To evaluate the development of active properties of the films related to the presence of phenolic compounds and their antioxidant capacity, film samples with the active extract were submitted to the extraction of phenolics with methanol and their quantification, evaluating the antioxidant capacity of the extracts after 1, 5, and 10 weeks of storage. Figure 4 shows the percentage of phenolics released from the different films (PLAes, PLAes-TPS, and PLAes-TPScf) with respect to their respective amount incorporated with the extract at different storage times and their DPPH radical scavenging capacity in terms of EC_50_ (amount of extract necessary to reduce by 50% the radical activity). After 1 week, all the active films released similar levels of incorporated phenolic compounds (about 75–85%). The lack of the total release of the phenolics incorporated suggests their partial degradation during the film thermoprocessing or their strong bonding to the PLA matrix. Nevertheless, the values of EC_50_ obtained at 1 week of storage, referred to as the equivalent extract in the films, were even lower than that obtained for the initial extract (6.3 mg extract·mg^−1^ DPPH). This suggests that new antioxidant compounds could be formed during the film thermoprocessing, such as caramelisation or Maillard compounds resulting from the extract sugar degradation that exhibit antioxidant activity [14]. After 5 or 10 storage weeks, a decrease in the released phenolics and antioxidant activity (EC_50_ increase) was observed for all films, according to the partial degradation of active compounds throughout time by hydrolysis, oxidation, or isomerisation reactions [40,41]. However, differences in the stability of active compounds could be observed for the different films without total coherence between phenolic content and radical scavenging capacity. After 10 weeks of storage, PLA monolayer and bilayer containing fibres released the highest phenolic amount, but PLA monolayer exhibited lower radical scavenging capacity than bilayers. Both bilayers had similar values of EC_50_ after 5 storage weeks and also after 10 weeks. Thus, different degrading kinetics of active compounds could be deduced for PLA monolayers and bilayers. This could be due to the progressive migration of active compounds to the starch layer with lower oxygen solubility, thus better preventing the oxidation of active compounds.

The obtained results indicate that there were changes in the physical properties of mono- and bilayers during storage, but these do not compromise their use as food packaging materials since the values reached were still in the range of initial properties that were validated in meat packaging with very good results [13]. Concerning the antioxidant capacity and phenolic content, a tendency to decrease was observed, but in the period tested, the materials still had a notable content of active compounds that can contribute to preserving packaged foods against oxidative and/or microbial spoilage, as it was observed with films conditioned for 1 week in meat preservation [13].

### 3.2. Biodegradability of the Mono and Bilayer Films

To analyse the film composting ability, respirometry analysis of CO_2_ and disintegration kinetics of the films under thermophilic composting behaviour were studied.

#### 3.2.1. Moisture Content, Thicknesses, and Elemental Carbon of the Films

Table 2 presents the different formulations of mono- and bilayer films with their corresponding equilibrium moisture content at 53% RH, thickness, and percentage of carbon, which affect the disintegration and biodegradation kinetics of the films. Starch monolayers with and without cellulose fibres showed the highest moisture content values (~8%), followed by bilayers (6.6–6.9%) and PLA monolayers (0.7–0.9%), which showed a much lower moisture content in accordance with their hydrophobic nature. The moisture content of bilayers was slightly higher than that predicted from the mass fraction of each polymer in the bilayer and their corresponding equilibrium moisture content (about 5%), which suggests a possible increase in the PLA water affinity in the bilayers due to the migration of glycerol from the starch sheet during the thermoadhesion step, as also deduced from the bilayer physical properties [13]. One of the factors that affect the polymer degradation kinetics is the hydration and diffusion of water through the polymer layers [42]. In this sense, the changes in the hydrophobic nature of PLA sheets will affect their ability to disintegrate or biodegrade when compared to PLA monolayers.

The starch monolayers exhibited higher thicknesses than PLA monolayers due to the higher starch mass per film (4 g vs. 3 g) and the lower fluency of starch during thermocompression, as observed by other authors [13]. The presence of fibres in the starch monolayers increased their thickness, which has been attributed to the glycerol interactions with the fibres that reduced its plasticising effect in the starch matrix, thus increasing the viscosity of its melt and reducing its flowability during thermocompression. The incorporation of the active extract did not produce significant changes in the thickness of the PLA films, but a decreasing trend was observed in line with the partial hydrolysis of the PLA chains by the hydrophilic RS extract [21]. This would reduce the length of the polymer chains, decreasing the viscosity of the melt and favouring its flowability during thermocompression. The bilayer films were less thick than expected from the sum of the thicknesses of the corresponding monolayers. This result can be associated with the radial flow of the polymer layers during the thermoadhesion step, conferring a lower thickness to each of them. Although there were no significant differences in the thicknesses of the different bilayers, there was a tendency for the thickness reduction of the samples with the extract. Likewise, during the thermoadhesion step, the interlayer migration of non-polymeric compounds, such as plasticisers (glycerol from starch and PEG1000 from PLA) or extract compounds from PLA sheet, can modify the flow behaviour of each layer with respect to that observed for isolated monolayers [13]. The different flow behaviour of starch and PLA sheets during the layer thermoadhesion could change their initial mass fraction in the bilayers, which could also explain the differences between their actual and predicted equilibrium moisture contents, as commented on above.

The carbon percentage of the different samples varies between 40 and 49%, depending on the molecular structure of the different components, their stoichiometric formula, and their proportion in the film. In particular, starch films, which have a higher proportion of oxygen in the molecule, have a lower proportion of carbon (40%), while PLA films present a higher percentage of carbon in the polyester molecule (49%). Coherently, the bilayer films have intermediate carbon percentages (44–45%).

#### 3.2.2. Compost Characteristics

The ripe compost used as inoculum in the disintegration and biodegradation tests showed a pH of 7.5, a total dry solid content (*DS*) of 77 ± 2%, and a volatile solid content (*VS*) of 91 ± 3%. Table 3 shows the characteristics of the synthetic solid residue (SSR) before and at the end of the composting process in each reactor, whether they contain or not the different films, in terms of the *VS* values and their percentage reduction (*R*, *%*) after the composting period. The *VS* values slightly decreased at the end of the composting process with respect to the initial value, indicating that the organic matter was being converted into CO_2_ by the microflora present in the compost. Nevertheless, the test was validated according to the standard procedure (ISO 20200, 2023), which establishes a standard deviation of the sample disintegration values (*D*_73_) of less than 10% and an R value greater than 30%. Additionally, throughout the test period, the compost underwent the colour changes described by ISO 20200 (2023), going from a lighter yellow (caused by the presence of sawdust) to a darker brown. The compost had a strong ammoniacal odour for the first week until it progressively disappeared, as stated in ISO 20200 (2023).

#### 3.2.3. Disintegration Test

The breakdown of the films into smaller fractions was determined by measuring the degree of disintegration (*D*_73_) of the films submitted to laboratory-scale composting conditions (58 ± 2 °C for 73 days) (Table 3). The disintegration values of the starch monolayers, with or without cellulose fibres, were similar (83–84%) and higher than those observed for both the control and active PLA monolayers (75 and 80%), as well as for all the bilayers (70–75%). This result is in line with the hydrophilic nature of starch, which allows bacteria to adhere quickly and consequently degrade its chemical structure. Although the presence of hydrophilic cellulose fibres could enhance film disintegration by promoting water absorption [43], this effect was not significant in hydrophilic starch films. Similar disintegration behaviour was reported by Pavon et al. [44] for starch films.

**Figure 5:**
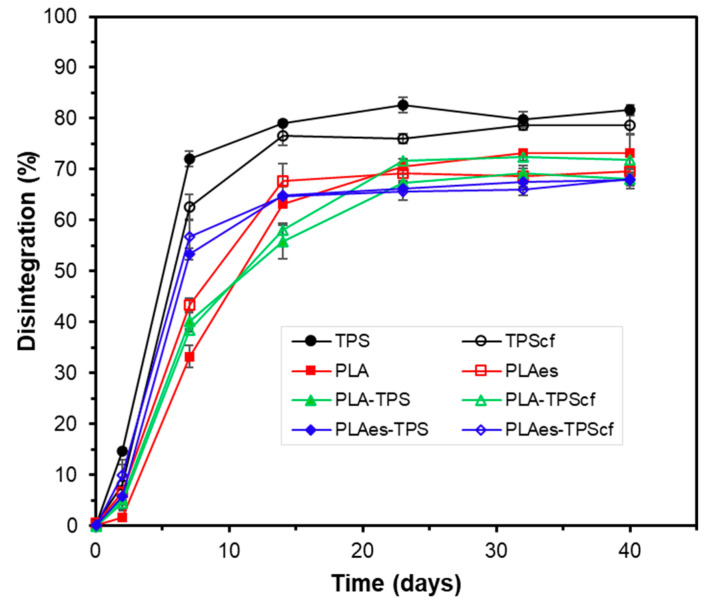
Development of sample disintegration as a function of time for the different monolayer (TPS, TPScf, PLA, PLAes) and bilayer (PLA-TPS, PLA-TPScf, PLAes-TPS, PLAes-TPScf) films. Mean values and standard deviations.

Kale et al. [45] described the disintegration of PLA under composting conditions initiated by the hydrolysis process of the polymer chains. The incorporation of RS extract slightly promoted the disintegration of PLA monolayers (PLAes) (*D*_73_: 80%) in comparison to the control PLA films that exhibited a *D*_73_ of 75%. As observed for the functional properties of the films, the presence of active extract promoted the degree of hydrolysis of the PLA chains, thus enhancing their disintegration. The lower disintegration values reached in the bilayers could be mainly attributed to their higher thickness since the disintegration process progresses from the surface to the inner part of the film by the progressive surface action of adhered biofilm [46]. Therefore, the lamination implies a reduction of the specific surface area for the action of microorganisms, and more time is required to reach the innermost polymer chains of the films, which explains the lowest *D*_73_ values of the bilayers. This tendency in D_73_ values was also observed in the disintegration kinetics shown in Figure 5, where practically asymptotic values in the film weight loss were reached from 20–25 days onwards for all samples. Mass fluctuations over the asymptotic period can be attributed to the different amounts of adhering compost particles or to the non-complete removal of the disintegrated particles from the mesh under the moist conditions of the samples that promote agglomeration and compaction of the powdered material in the mesh. The delayed disintegration of PLA monolayers in comparison to starch films was observed, as well as the effect of RS extract on promoting PLA disintegration. Likewise, bilayers with RS extract disintegrated faster than those without extract, whereas a non-significant effect was observed for RS fibres on the disintegration kinetics of bilayers. In contrast, the fibres slightly reduced the initial disintegration rate of starch monolayers, probably due to their reinforcement effect.

**Figure 6:**
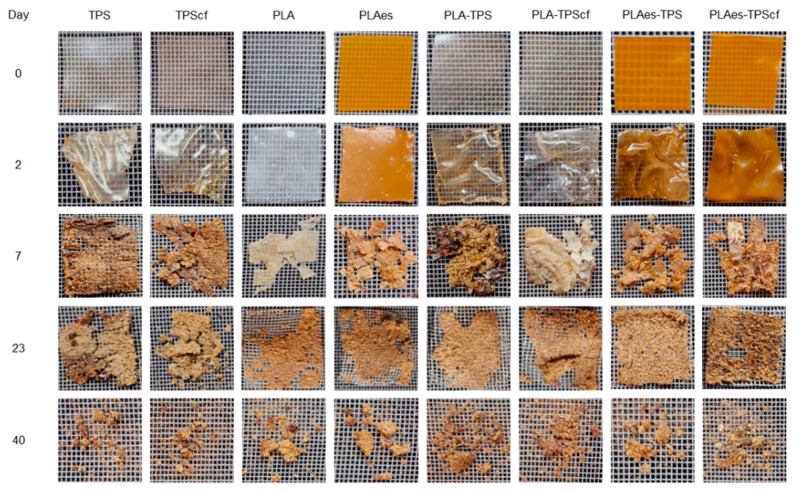
Photographs of the monolayer (TPS, TPScf, PLA, PLAes) and bilayer (PLA-TPS, PLA-TPScf, PLAes-TPS, PLAes-TPScf) films at different composting times.

Figure 6 shows the visual appearance of the samples at different composting times. It is remarkable that the films were already significantly disintegrated after 20–25 days of composting, as can be deduced from Figure 5 and Figure 6. In fact, the visual appearance of the samples at 40 days corresponded to particles with a certain degree of agglomeration due to the high moisture content in the reactor and the protection/mechanical action of the mesh. Small differences were observed between the different monolayer and bilayer films. Therefore, although all samples showed a disintegration behaviour that meets the requirements set out in the Standard to be labelled as compostable, differences in behaviour were observed, depending on the composition and the different mechanisms involved in the disintegration of each layer. Specifically, the presence of extract accelerated the initial disintegration rate of PLA monolayers and bilayers with and without fibres, with the disintegration rate of the latter being higher than that of PLA monolayers. This is consistent with the thermal effects during the layer adhesion that could enhance the hydrolytic action of PLA and starch layers due to the interlayer diffusion of bound water from the starch layer and of the extract hydrolytic compounds (e.g., phenolic acids) from the PLA layer. Therefore, despite the greater thickness of the bilayers, their disintegration occurred within a similar time period to that of the monolayers, both being slightly affected by the presence of RS fibres or active extracts.

#### 3.2.4. Biodegradation Test

**Figure 7:**
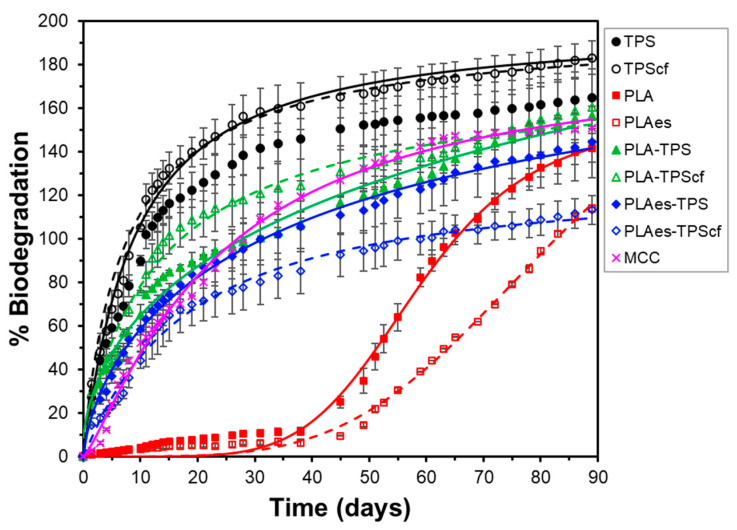
Biodegradation kinetics of CMC, monolayer (TPS, TPScf, PLA, PLAes), and bilayer (PLA-TPS, PLA-TPScf, PLAes-TPS, PLAes-TPScf) films throughout the composting time. Experimental data (symbols) and Hill’s fitted model (lines).

The biodegradation of the films was also analysed under thermophilic composting conditions at a laboratory scale for 90 days at 58 °C, following ISO 14855, through respirometry analyses. The theoretical maximum amount of carbon dioxide that can be produced from the total biodegradation of the samples was calculated from their carbon content (elemental analysis). The percentage of biodegradation of the samples at each time was determined by dividing the accumulative CO_2_ determinations (corrected with the blank values) by the maximum theoretical value (Equation (8)). The biodegradation profile of microcrystalline cellulose (MCC) was also analysed as a reference sample. Figure 7 shows the development of the biodegradation percentage as a function of composting time for the different mono- and bilayer films and the CMC reference. To validate the quality of the inoculum used in the biodegradation test, the ISO 14855 standard establishes that the degree of biodegradation of the reference material must be higher than 70% after 45 days. As can be seen in Figure 7, the degree of biodegradation of the reference material (CMC) was 121% after 45 days of composting, which validates the experimental data.

All biodegradation curves exhibited the typical sigmoid curve reported by other authors [47] for different polymers resulting from the different steps of the process. Different factors, such as crystallinity degree, side-chain length, shape or surface morphology, and properties of the biodegradation medium (temperature, UV exposure, nutrient levels, mechanical forces, bacteria population, pH, and oxygen levels), influence the biodegradation rate at any specific time, which introduces complexity in the process. Despite its complexity and iterative concurrence of the different phases, a simplified biodegradation process has been conceptualised with three key steps with their respective rates [46]: (1) the formation of biofilm, which is a complex association of microbes formed from surface-associated microbial cells embedded in a self-produced extracellular polymeric matrix [48], requiring adaptation time (lag time) [49], (2), hydrolytic bond cleavage of the polymer by the extracellular depolymerases, leading to the formation of oligomers, dimers, and monomers at a determined rate, (3) the bioassimilation of small molecules by the cells for either the growth and reproduction or mineralisation step, where the resulting products increased cell biomass and generation of compounds such as CO_2_ (in aerobic conditions) and water.

Starch monolayer films presented the highest degradation rate without the notable influence of the cellulose fibres. In contrast, PLA monolayer films showed an induction phase with almost zero biodegradation rate, followed by a period with quantitative CO_2_ generation, starting at about 35 days, where the presence of extract had an inhibitory effect, significantly reducing CO_2_ generation. On the other hand, the bilayer films showed no inhibition period, although their biodegradation rate was lower than that of the starch monolayers. The presence of fibres or extract in the bilayers significantly affected their biodegradation behaviour. The incorporation of extract always reduced the degradation rate of the active bilayers, but this effect was affected by the presence or absence of cellulose fibres in the starch layer. Without fibres, negligible differences in the biodegradation pattern of the bilayers due to the presence of extract were observed. Nevertheless, a marked effect of fibres in the starch layer was observed on the inhibitory effect of the extract on biodegradation. The RS extract exhibits antimicrobial properties [10,13,14,21], and the presence of antimicrobials in the film could affect the biofilm adhesion and its microbial population on the film surface, which would affect the enzyme hydrolytic action responsible for chain scission and polymer degradation [49].

PLA degradation in compost involves a long lag period (Figure 7) since the process starts at the surface level with the hydrolysis of the polymer chains induced by the water diffusion into the materials, causing a random breakdown of the polymer to form oligomers and lactic acid [50]. Subsequently, once the molecular weight reaches approximately 10,000–20,000 D, stable biofilm formation and enzymatic degradation take place, leading to the formation of carbon dioxide, water, and humus [45]. In contrast, starch degradation is faster caused by enzymes released by adhered microorganisms through a surface erosion mechanism, gradually spreading to the entire bulk [46]. For the biodegradation of the bilayers, the superposition of both PLA and starch degradation mechanisms will occur on each side of the laminate, giving rise to an intermediate overall behaviour. Likewise, the biodegradation rate of bilayers can be influenced by the interlayer migration of low molecular weight compounds. In particular, the low effect of the extract on the biodegradation pattern of the bilayers without fibres is remarkable, which indicates that the bioactive compounds are less available to the biofilm microorganisms and, therefore, do not have a notable effect on CO_2_ generation. This lower availability could be explained by their migration into the starch layer due to the higher chemical affinity with this polymer and the subsequent dilution effect, even affected by their potential binding to the OHs in the starch matrix, thus attenuating its antimicrobial effect. In contrast, this migration could be reduced by the presence of cellulose fibres in the starch layer, which has a previously observed glycerol sequestering action [14], reducing molecular mobility and diffusion properties of the matrix. The lower migration of the extract compounds to the starch layer would give rise to higher concentrations in the PLA layer, which would have a greater impact on the microbial biofilm.

Apart from the behavioural differences, every film reached maximum biodegradation values above 100% (*B_max_* in Table 4) after a certain composting time, which is attributed to the so-called “priming effect”. This effect occurs when the compost inoculum in the reactors of the samples produces more CO_2_ than in the blank reactors. In this case, the microflora is overstimulated by the small molecules that are released into the medium as a consequence of polymer degradation [51]. Table 4 shows the parameters of Hill’s model fitted to the experimental curves, where the values of the time necessary to reach 50% total biodegradation (*k*), the curvature coefficient (*n*), and the asymptotic or final value of biodegradation (*B_max_*) can be observed. The values obtained are coherent with the biodegradation rates previously discussed for each formulation. Starch monolayer films, with and without fibres, reach 50% biodegradation in about 20 days, while PLA monolayer films require 84 (PLAes) or 59 (PLA) days, depending on whether they have active RS extract. For the bilayers, this period ranges from 35 to 45 days, depending on whether they contain cellulosic fibres and/or active extract. The bilayers with extract biodegraded to 50% in 46 days when no fibres were present, whereas they needed 39 days when they contained cellulose fibres. On the other hand, the bilayers without active extract biodegrade to 50% in fewer days (35–38), depending on the absence or presence of cellulose fibres. These time values were also affected by the maximum value of CO_2_ generation (*B_max_%* in Table 4) for each film.

Therefore, all films can be considered compostable since they reach 100% biodegradation before 90 days of assay. Although PLA showed a slower degradation rate, its bilayer assembly with starch reduced the lag period for biodegradation. This can be explained by its contact with the starch layers and the migration of low molecular weight compounds between the polymer matrices, which contributed to accelerating the overall biodegradation of the bilayer. The aforementioned phenomena could promote the hydrolytic stage of PLA prior to CO_2_ generation [45], contributing to the faster progress of the degradation process.

## 4. Conclusions

Starch and PLA monolayers and bilayers with and without active extract and cellulose fibres from rice straw showed changes in their mechanical and barrier properties to water vapour and oxygen during storage at 25 °C and 53% relative humidity. The most notable changes occurred in the starch monolayers due to the starch retrogradation, which involved an increase in stiffness and loss of extensibility of the films, with an increase of WVP at the longest times. However, these changes were more attenuated in the bilayers that gained water vapour and oxygen barrier capacity during storage, maintaining the values of the different properties close to the initial range. The content of phenolic compounds and antioxidant capacity of potentially active films decreased slightly but remained at acceptable levels for consideration as active materials, with the antioxidant capacity better maintained in the bilayers. Therefore, the PLA–starch bilayers with and without active extract and cellulosic fibres from rice straw stables throughout 10 storage weeks maintain their properties reasonably well during this period, which allows their commercialisation as food packaging materials.

On the other hand, all monolayer and bilayer films were composted within 90 days. The bilayer assembly enhanced the biodegradation of PLA, reducing the lag period of PLA monolayers. The active extract reduced the biodegradation rate of PLA monolayers and bilayers but did not limit the total biodegrading of the materials within the time established in Standard. Therefore, PLA–starch laminates, with and without active extracts and cellulose fibres from rice straw can be considered as stable and compostable materials for food packaging applications.

## Figures and Tables

**Table 1:** Colour coordinates (L*, C_ab_*, and h_ab_*) of mono- and bilayer films stored at 25 °C and 53% relative humidity for 1, 5, and 10 weeks.

Film	L*			C_ab_*			h_ab_*		
	S1	S5	S10	S1	S5	S10	S1	S5	S10
PLA	90.7 ± 0.2 ^a,1^	90.8 ± 0.1 ^a,1^	90.1 ± 0.3 ^a,1^	2.5 ± 0.13 ^e,1^	2.42 ± 0.12 ^e,1^	2.33 ± 0.17 ^e,1^	99.6 ± 0.8 ^a,1^	100.1 ± 0.21 ^a,1^	100.7 ± 0.5 ^a,1^
PLAes	67.6 ± 0.7 ^f,1^	68.3 ± 1.7 ^f,1^	66.2 ± 1.2 ^f,1^	34.57 ± 0.7 ^a,1^	32.85 ± 3.0 ^a,1^	33.72 ± 1.20 ^a,1^	77.1 ± 0.3 ^f,1^	79.0 ± 1.0 ^f,1^	78.5 ± 0.5 ^f,1^
TPS	88.5 ± 0.1 ^b,1^	89.0 ± 0.1 ^b,1^	87.8 ± 0.9 ^b,1^	7.57 ± 0.10 ^d,1^	7.3 ± 0.88 ^d,1^	7.12 ± 0.33 ^d,1^	92.5 ± 0.1 ^b,1^	94.7 ± 0.7 ^b,1^	95.4 ± 0.8 ^b,1^
TPScf	88.1 ± 0.2 ^b,1^	87.8 ± 0.3 ^b,1^	88.3 ± 0.7 ^b,1^	8.25 ± 0.35 ^cd,1^	8.7 ± 0.48 ^cd,1^	8.2 ± 0.28 ^cd,1^	92.6 ± 0.2 ^b,1^	92.4 ± 0.4 ^b,1^	92.1 ± 0.6 ^b,1^
PLA-TPS	87.3 ± 0.3 ^b,1^	88.4 ± 0.5 ^b,1^	87.9 ± 0.7 ^b,1^	7.58 ± 0.11 ^d,1^	7.3 ± 1.22 ^d,1^	7.1 ± 1.31 ^d,1^	92.2 ± 0.3 ^b,1^	95.1 ± 1.2 ^b,1^	93.1 ± 2.1 ^b,1^
PLA-TPScf	86.0 ± 0.5 ^c,1^	87.2 ± 0.3 ^c,1^	87.8 ± 0.4 ^c,1^	9.50 ± 0.31 ^c,1^	9.79 ± 0.36 ^c,1^	9.34 ± 0.22 ^c,1^	90.9 ± 0.4 ^c,1^	92.7 ± 0.5 ^c,1^	91.4 ± 0.5 ^c,1^
PLAes-TPS	76.9 ± 1.2 ^d,1^	74.5 ± 3.0 ^d,1^	75.4 ± 1.6 ^d,1^	27.92 ± 2.06 ^b,1^	25.83 ± 2.98 ^b,1^	25.14 ± 1.28 ^b,1^	83.4 ± 0.7 ^d,1^	80.4 ± 1.6 ^d,1^	80.7 ± 0.7 ^d,1^
PLAes-TPScf	72.2 ± 0.9 ^e,1^	72.0 ± 2.0 ^e,1^	73.1 ± 1.1 ^e,1^	34.41 ± 1.22 ^a,1^	34.32 ± 2.68 ^a,1^	34.24 ± 2.47 ^a,1^	81.0 ± 0.6 ^e,1^	81.0 ± 1.4 ^e,1^	80.5 ± 0.2 ^e,1^

Different letters in the same column indicate significant differences between the formulations by the Fisher test (α = 0.05). Different numbers in the same row indicate significant differences between the storage times for each formulation by the Fisher test (α = 0.05).

**Table 2:** Moisture content (MC), thickness, and content of elemental carbon (C %) of the sample films. Mean values and standard deviation.

Sample	Moisture Content (%)	Thickness (µm)	C (%)
TPS	8.3 ± 0.2 ^a^	0.171 ± 0.026 ^bc^	40.3 ± 0.1
TPScf	8.0 ± 0.6 ^a^	0.185 ± 0.025 ^b^	40.2 ± 0.2
PLA	0.7 ± 0.1 ^c^	0.146 ± 0.008 ^c^	48.7 ± 0.1
PLAes	0.9 ± 0.1 ^c^	0.144 ± 0.007 ^c^	49.0 ± 0.5
PLA-TPS	6.9 ± 0.3 ^b^	0.278 ± 0.033 ^a^	45.0 ± 0.4
PLA-TPScf	6.7 ± 0.4 ^b^	0.273 ± 0.012 ^a^	43.8 ± 0.1
PLAes-TPS	6.7 ± 0.3 ^b^	0.269 ± 0.013 ^a^	44.7 ± 0.1
PLAes-TPScf	6.6 ± 0.1 ^b^	0.262 ± 0.007 ^a^	44.8 ± 0.2
MCC	-	-	42.2 ± 0.1

Different letters in the same column indicate significant differences between the formulations by the Fisher test (α = 0.05).

**Table 3:** Volatile solids (*VS*) (g volatiles·100 g^−1^ compost *DS*) before and after the composting period, the percentage change in these values with respect to the initial ones (R %) and film disintegration percentage at 73 composting days (*D*_73_). Mean values and standard deviations.

Sample	*VS* (g·100 g^−1^ DS)		*R* (%)	Desintegration *D*_73_ (%)
	Pre-Composting	Post-Composting		
SSR	90.6 ± 1.8	88.3 ± 2.1	43.2	-
TPS	-	85.1 ± 1.2	48.1	84 ± 1
TPScf	-	83.1 ± 3.4	45.1	83 ± 1
PLA	-	82.4 ± 1.5	51.3	75 ± 1
PLAes	-	87.3 ± 2.7	49.2	80 ± 2
PLA-TPS	-	85.4 ± 1.5	48.2	75 ± 2
PLA-TPScf	-	83.4 ± 2.7	53.2	74 ± 3
PLAes-TPS	-	80.4 ± 2.4	47.6	70 ± 2
PLAes-TPScf	-	83.4 ± 1.2	49.2	71 ± 3

SSR: synthetic solid residue.

**Table 4:** Hill’s parameters: *n*, *k* (the time needed for 50% of *B_max_* to occur), and *B_max_* (percentage of biodegradation at the infinite time) for the different films and microcrystalline cellulose (MCC, reference) and *R*^2^ (correlation coefficient for the fitted model).

Sample	*n*	*k* (Days)	*B_max_* (%)	*R* ^2^
MCC	1.2	23.6	185	0.995
TPS	1.0	20.1	198	0.994
TPScf	0.9	22.3	196	0.996
PLA	5.6	58.5	155	0.992
PLAes	4.2	84.0	125	0.992
PLA-TPS	0.4	35.2	163	0.998
PLA-TPScf	0.9	38.4	172	0.985
PLAes-TPS	0.7	45.8	147	0.996
PLAes-TPScf	1.0	39.4	129	0.990

## Data Availability

Data are contained within this article.
